# Physiological, Behavioral and Maternal Factors That Contribute to Size Variation in Larval Amphibian Populations

**DOI:** 10.1371/journal.pone.0076364

**Published:** 2013-10-15

**Authors:** Robin W. Warne, Adam Kardon, Erica J. Crespi

**Affiliations:** Biology Department, Vassar College, Poughkeepsie, New York, United States of America; Clemson University, United States of America

## Abstract

Size variance among similarly aged individuals within populations is a pattern common to many organisms that is a result of interactions between intrinsic and extrinsic traits of individuals. While genetic and maternal effects, as well as physiological and behavioral traits have been shown to contribute to size variation in animal populations, teasing apart the influence of such factors on individual growth rates remain a challenge. Furthermore, tracing the effects of these interactions across life stages and in shaping adult phenotypes also requires further exploration. In this study we investigated the relationship between genetics, hatching patterns, behaviors, neuroendocrine stress axis activity and variance in growth and metamorphosis among same-aged larval amphibians. Through parallel experiments we found that in the absence of conspecific interactions, hatch time and to a lesser extent egg clutch identity (i.e. genetics and maternal effects) influenced the propensity for growth and development in individual tadpoles and determined metamorphic traits. Within experimental groups we found that variance in growth rates was associated with size-dependent foraging behaviors and responses to food restriction. We also found an inverse relationship between glucocorticoid (GC) hormone levels and body mass and developmental stage among group-reared tadpoles, which suggests that GC expression plays a role in regulating differing within-population growth trajectories in response to density-dependent conditions. Taken together these findings suggest that factors that influence hatching conditions can have long-term effects on growth and development. These results also raise compelling questions regarding the extent to which maternal and genetic factors influence physiological and behavioral profiles in amphibians.

## Introduction

Size variation among similarly aged individuals is a characteristic common to many animals [1], [2]. Variation in growth patterns among individuals has been associated with biotic and abiotic factors that include population density, resource availability and environmental heterogeneity [3]–[5]. In addition intrinsic factors, such as genetic differences and maternal effects, as well as physiological and behavioral traits can influence how individuals differentially respond to environmental conditions. The interactions between such intrinsic and extrinsic factors likely determine the degree of size variation among evenly aged cohorts in animal populations. Because body size is fundamental to organismal biology, such interactions on growth patterns not only influence individual performance and fitness but also impact population dynamics. However, teasing apart the influence of such intrinsic and extrinsic factors on individual growth rates remain a challenge. Furthermore, tracing the effects of these interactions across life stages and in shaping adult phenotypes also requires further exploration.

Initial size at birth or hatching, for example, can have long-term effects through imposing the need for catch-up growth, which can shape adult physiology and behavior through its effects on resource allocation to growth at the potential expense of other processes [6], [7]. Size at hatching and these subsequent resource allocation dynamics have been associated with maternal effects such as egg size [8], [9]. The influence of maternal effects on individual growth rates is likely influenced by environmental factors that include population density, because with increased population density comes intensification of competition [1], [5]. At the center of these dynamics is obviously how individuals vary in their physiological and behavioral responses to population density and other potentially stressful environmental conditions.

Larval frogs (i.e., tadpoles) provide a biologically unique and ecologically rich system to study how individual variation in growth is related to physiological function and behavior. Tadpoles can exhibit great variation in body size within populations that results from highly varied growth and development rates among individuals, traits that determine timing and size at metamorphosis [10]–[14]. As in other animals populations, when resources are restricted and/or densities are high the variance in growth rates increases within populations, which suggests an increase in competitive interactions [5], [15]–[17]. While individual responses to such environmental conditions is central to Wilbur and Collins [14] seminal model of complex life histories [13], [18]–[20], the physiological and behavioral mechanisms underlying individual variation among tadpoles has not been resolved. There is evidence that tadpoles are sensitive to and exhibit physiological and behavioral responses to density dependent interactions [21], [22]. Tadpoles use visual, tactile, and chemical cues to detect other tadpoles around them [22], [23]. Tadpoles may also emit chemicals that can inhibit the growth of others [15]. Studies have also suggested that inter- and intraspecific exploitative competition is present in anuran tadpoles under low resource or high density conditions [15]–[17], [24]. Furthermore, in some anuran species aggregations of tadpoles among kin can increase growth rates and body size, especially for smaller tadpoles within a group [25]–[28]. These findings suggest that intrinsic factors and individual behavioral and physiological traits likely regulate growth and size variance in tadpole populations.

Variance in growth rate may reflect behavioral and physiological differences in how tadpoles respond to density dependent environmental conditions. As in other vertebrates, the hypothalamo-pituitary-interrenal neuroendocrine stress axis (HPI axis) likely plays a role in mediating growth patterns associated with density dependent responses in tadpole populations [29]. In laboratory experiments, both elevated corticotropin-releasing factor, the hypothalamic regulator of the HPI axis, and chronically elevated glucocorticoid levels directly inhibit food intake and suppress growth in anuran tadpoles [30]–[32]. Furthermore, a reduction in food resources or an elevation in perceived or actual densities of conspecific tadpoles, are associated with increased corticosterone (CORT) levels, the dominant glucocorticoid in amphibians [22], [31], [33]. In natural populations, Belden et al. [34] also found that smaller wood frog (*Lithobates sylvaticus*) tadpoles had higher baseline and stress-induced CORT levels relative to larger individuals, however, the history and development stages of these tadpoles were not known. Taken together, these findings support the hypothesis that variation in HPI axis activity among tadpoles in relationship to population density influences growth patterns and metamorphic traits.

In this study we investigated the relationships between family (i.e. egg clutch), hatching patterns, foraging behaviors, stress axis activity and variance in growth among larval wood frogs (*Lithobates sylvaticus*). First, we examined how family identity and hatching time influence individual growth and development rates. We then explored how individual behaviors influence growth variance among tadpoles. Here, we used visible elastomer tags to make longitudinal observations of individual behaviors within experimental groups. Last, we investigated the relationship between CORT levels and growth rates of tadpoles raised since hatching in groups.

## Materials and Methods

### Animal collection and housing

Four egg masses of the same developmental stage were collected within 1–2 days of being laid from two separate ponds located within the Vassar College Ecological Preserve (NY DEC permit #1293). Because each mass was collected from distant locations within ponds, we assume they were laid and fertilized by different parents, thus represent different families. Egg masses were individually housed in 19-liter aquaria with air stones at 18°C in the laboratory. After all the tadpoles hatched, those that were not immediately utilized in experiments were mixed and housed in 200-liter outdoor containers (∼300 tadpoles/tank) with leaf litter and fed Timothy Hay rabbit pellets *ad libitum*, and were placed into experiments as described below. Experiments were conducted under the approval of the Vassar College Institutional Animal Care and Use Committee (Protocol #10–04B).

### Family and hatching order effects on individual growth rates

To examine the degree of variation in individual propensities for growth, hatchlings were individually reared through metamorphosis in the absence of group interactions to estimate the contribution of family (i.e., genetic and maternal effects) on variance in growth rates. In order to isolate the effect of hatching date as a factor differentiating tadpoles within a family, five tadpoles/day were collected from each clutch (n = 4) during four sequential hatching days (all tadpoles in a clutch hatched within five days), for a total of 80 tadpoles (20 hatchlings/family; 20 hatchlings/hatching date). Tadpoles were housed individually in 500 mL of water and fed 0.1 g of pulverized Timothy Complete pellets/week for 4 weeks and then 0.2 g/week thereafter. Housing containers were kept on a 12L∶12D light cycle at 66–68°F in the Vassar College animal care facility. Tadpoles were weighed at 8, 16, 24, and 52 days post-hatching, and at metamorphosis (Gosner stage –41) [35]; snout-vent length and time to metamorphosis were also recorded. Logistic by weight growth models were then fit to these weight measurements using JMP 10.0 to quantify individual growth rates [36], [37].

### Behavioral observations in experimental groups

**Figure 1:**
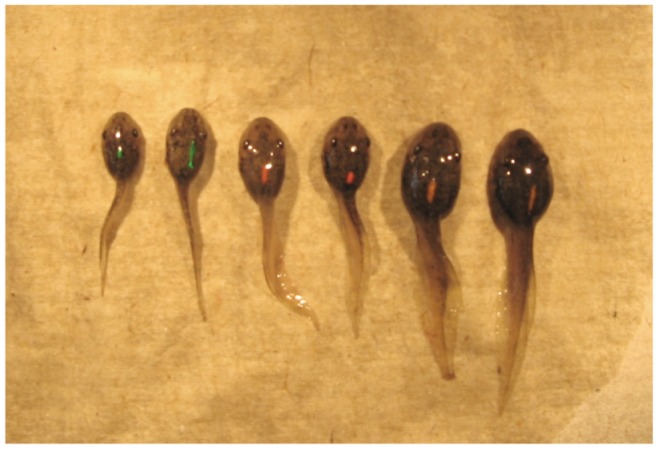
Wood frog tadpoles marked with subcutaneous elastomer tags for individual identification in behavioral observations.

The goal of this experiment was to examine behavioral effects on growth trajectories of tadpoles that were of similar body mass and developmental stage. Tadpoles at Gosner stage 29 were collected from stock tanks of mixed families and placed into six replicate experimental groups (n = 8 tadpoles/group). In each group, tadpoles were matched to minimize body size variance at the start of the experiment. In order to observe individuals over time, a unique color combination of elastomer tags (Northwest Marine Technology, Inc.) [38] was injected subcutaneously along the midline of the dorsal fatty tissue between the brain case and tail of each tadpole (Fig. 1) while under anesthesia (0.01% benzocaine solution). Once tadpoles revived and showed no adverse effects of the procedure, each group was placed in a 19-liter aquarium in an indoor animal room kept at room temperature with a 12L∶12D photoperiod. The aquarium were filled with 18 liters of water for a density of 1 tadpole/2.25 L, which is similar to moderate densities that can be found in nature and in the range of densities in which behavioral differences have been noted [5], [39], [40]. Tadpoles were fed 0.25 g of pulverized Timothy Complete pellets four times per week, with weekly water changes. These groups grew for five weeks then all individuals were euthanized, staged and weighed.

We conducted behavioral measures in two different assays conducted between 2–3 weeks after groups were established. First, we recorded the time each tadpole spent foraging, swimming, or resting using an Excel-based logging program [30] during a three-minute observation period between 900–1500 hr. Three replicate observations were conducted for each tadpole within a 7-day span, and the time spent for each behavior was summed over the three observations. In a second behavioral assay, we removed food from the tanks for 24 hrs, and then placed a single food pellet in the center of the tank. The time that each tadpole took to approach the food was recorded in order to measure an individual's affinity to eat, a proposed form of competition.

### Stress axis activity and growth trajectories in experimental groups

This experiment was designed to determine the relationship between CORT content and size variation within groups raised together from hatching. Tadpoles from each egg clutch (as above) were collected on a single day of hatching, mixed and randomly assorted into six groups of eight tadpoles fed 0.25 g Timothy Complete pellets two times per week. Each group was housed in 19-liter aquaria for 8 weeks. By allowing the tadpoles to develop together from hatching, we were able to associate CORT levels to growth and development rates. This protocol, however, prevented us from observing individual behaviors because tadpoles were too small at the start of the experiment for elastomer tags (see above). At the conclusion of the experiment, all tadpoles were frozen for whole-body CORT analysis using a radioimmunoassay (RIA) procedure described in Warne et al. [41]. Two RIA assays were run, with an inter-assay coefficient of variation (CV) of 8%, and mean intra-assay CV of 2%.

### Statistical analysis

For the individual growth rate experiment, the effects of family (egg clutch) and hatching time and their interaction on logistic growth rates were analyzed with general linear models (GLM); here hatching mass was used as a covariate because mass at hatching may influence growth rates [37]. GLM was also used to test for the effects of these factors on hatching mass and mass at metamorphosis. To meet assumptions of normality, growth rates and body mass were log transformed in all analyses, and post-hoc comparisons were conducted using Tukey-Kramer's HSD tests. To determine the effects of family and hatching time on metamorphic timing, we used Kaplan-Meier plots [42] to plot the probability of metamorphosis for each hatch day, based on the distribution of days until metamorphosis since hatching. The Fit Parametric Survival function in JMP^®^ 10.0 was used to determine differences in the likelihood of metamorphosis using an underlying lognormal distribution of metamorphic times. For the behavioral assays, random-effects GLM with tank as a random factor were used to test for associations between specific growth rate (ln[mass_2_/mass_1_]/[t_2_-t_1_]), final body mass and mean foraging time, as well as between time to find food and final body mass. Here we used specific growth rates rather than logistic growth models (as above) because only initial and final weights were measured. To better describe the effects of each tank, we also calculated Pearson correlations between the behaviors and final body size or growth rate. For the CORT experiment, a random-effects model was also used with tank as a random factor to measure the relationship between CORT content, final body mass, developmental stage and family. Note that because these are random-effects models the degrees of freedom are estimates. All analyses were conducted using JMP^®^ 10.0 [43].

## Results

### Family and hatching order effects on individual growth rates

**Figure 2:**
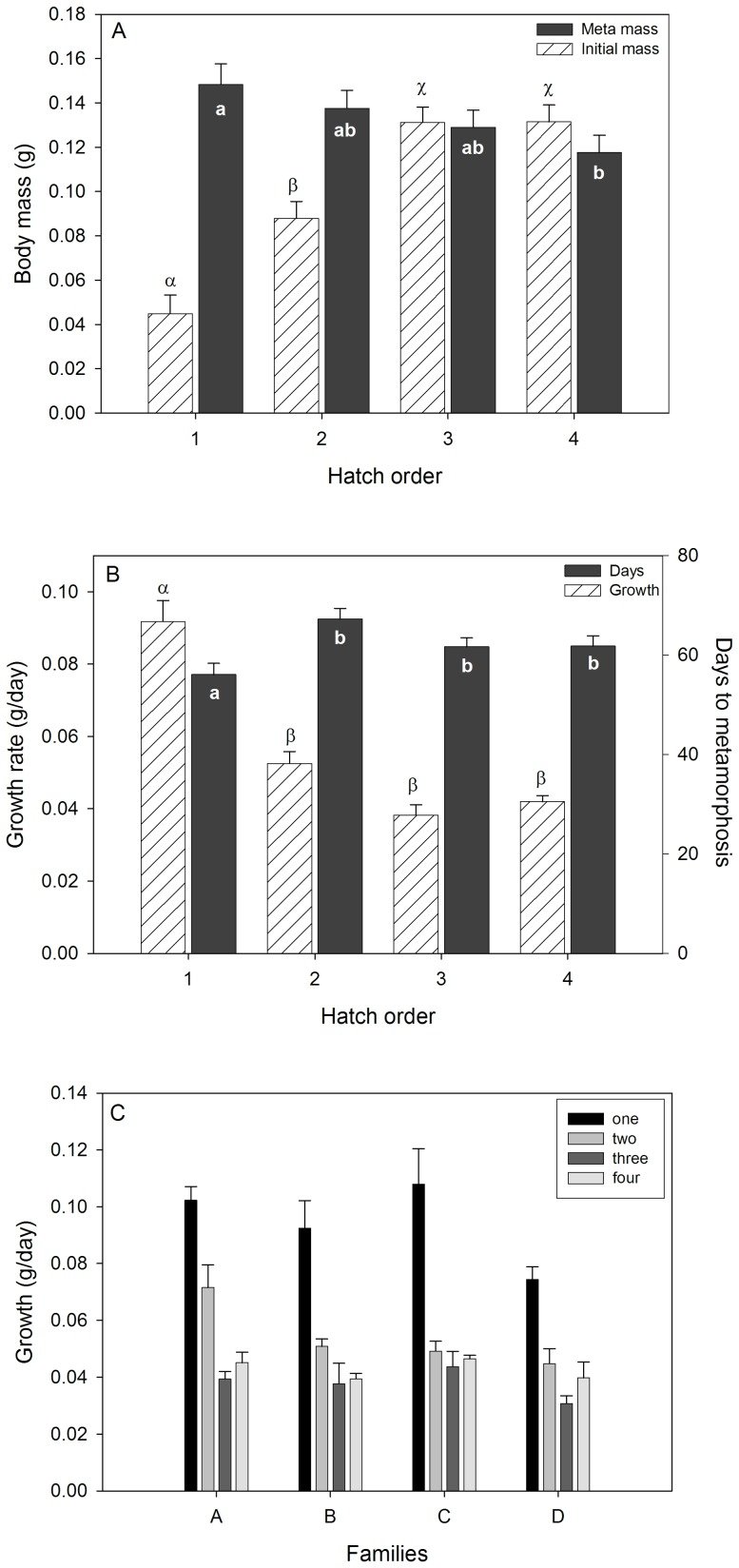
The effects of hatching order and family body mass, metamorphic traits and growth rates. Hatching order influenced body mass at hatching and at metamorphosis for tadpoles reared in isolation (A). Hatch order also influenced growth and developmental rates (B). Family (i.e. egg clutch) did not influence growth rates (C). Differing letters or symbols denote significant differences between hatch orders for each trait (Tukey HSD test, P<0.05).

Hatching order was strongly associated with tadpole body mass at hatching (Figure 2A; *F*_3,50_ = 52.6, *P*<0.0001; Table 1) and at metamorphosis (*F*_3,50_ = 3.8, *P* = 0.02), and was inversely related to growth rates (Figure 2B; *F*_3,43_ = 4.4, *P* = 0.009). Tadpoles hatching on days 1 and 2 were smaller at hatching but grew faster than later hatching tadpoles (hatch day 1– Tukey HSD P<0.05), resulting in larger body sizes at metamorphosis. Mass at hatching also influenced growth rates (Table 1; *F*_1,43_ = 4.9, *P* = 0.03) and had an interaction with hatch order (*F*_3,43_ = 3.86, *P* = 0.02). While body mass or growth rate did not vary among families, there was an interaction between family and hatching order on growth rates (Table 1; Figure 2C). In all families, the tadpoles hatching on the first day grew the fastest, however growth rates did not vary consistently across the other hatching days. Hatch order also had a pronounced effect on development and timing of metamorphosis, such that the first hatched tadpoles metamorphosed on average at 56±2.1 days after hatching (Figure 2B), which was up to 9 days earlier than tadpoles that hatched on day 4 (65±1.7 days); differences between hatch order probability curves for metamorphosis (not shown) were significant (likelihood ratio χ^2^ = 28, *P*<0.0001). Family also influenced metamorphic timing (χ^2^ = 26.97, *P*<0.0001), and there was a family by hatch order interaction (χ^2^ = 19.8, *P* = 0.02). This family influence was largely driven by family D that exhibited the slowest growth rates (Figure 2C) and metamorphosed at 67±1.5 days after hatching, which was 7 days later on average than the other families.

**Table 1:** F-statistics and P-values for general linear models of hatch order and family associations hatching mass, mass at metamorphosis and logistic growth rates.

	Hatching mass			Metamorph mass			Growth Rates		
Source	DF	F Ratio	P > F	DF	F Ratio	P > F	DF	F Ratio	P > F
Hatch order	3, 50	52.59	**<0.0001**	3, 50	3.83	**0.015**	3, 43	4.37	**0.009**
Family	3, 50	2.13	0.108	3, 50	1.09	0.362	3, 43	1.67	0.187
Family*Hatch order	9, 50	1.54	0.161	9, 50	1.20	0.318	9, 43	2.65	**0.016**
Log Hatching Mass							1, 43	4.87	**0.033**
Hatch order*Hatching Mass							3, 43	3.86	**0.016**
Family*Hatching Mass							3, 43	0.57	0.640

### Behavioral observations in experimental groups

**Figure 3:**
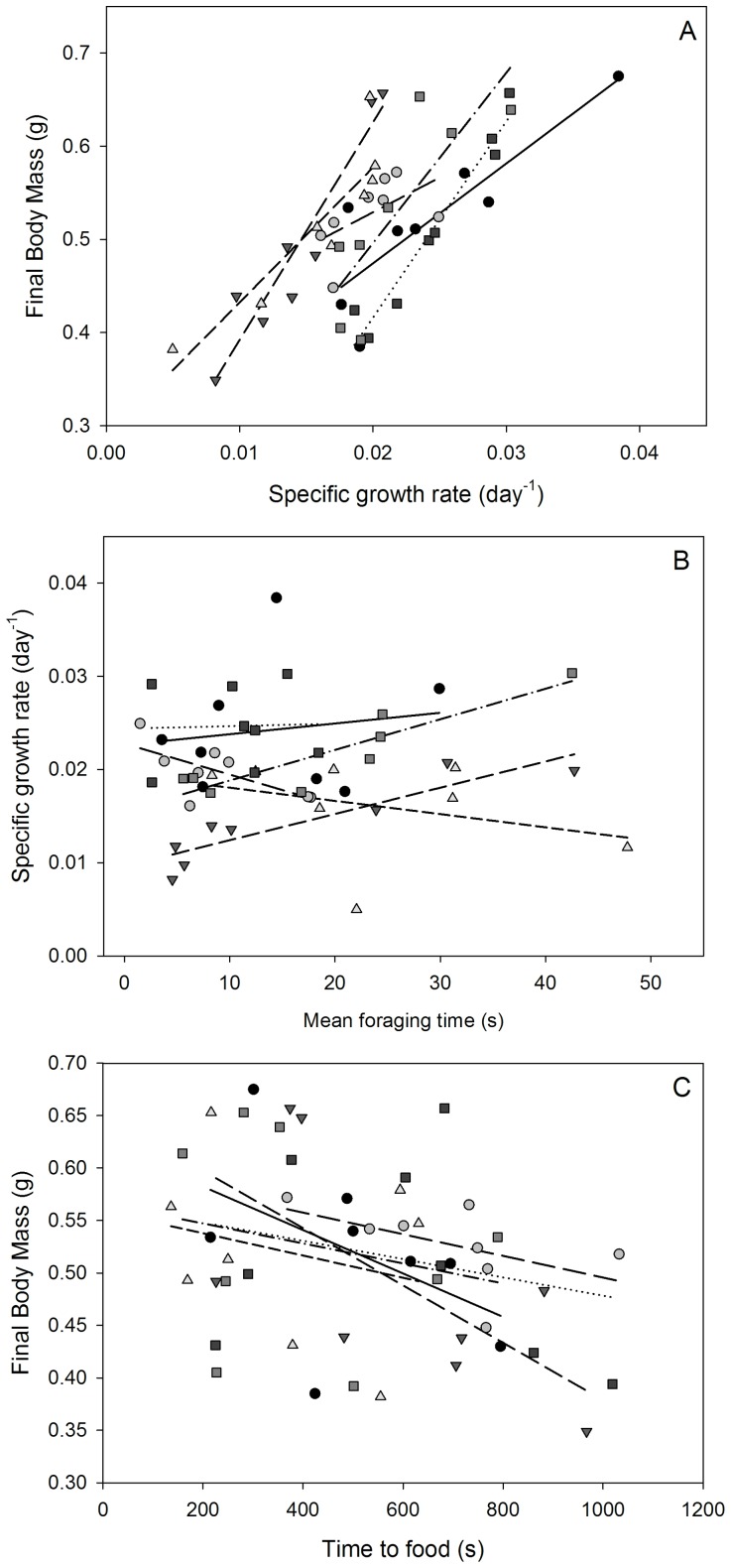
Behavioral analysis of initially similarly sized tadpoles that were set into groups at a premetamorphic stage (Gosner stage 29). Growth rate was associated with the final size of tadpoles after 5(A) and the mean time that tadpoles spent foraging was associated with growth rates (B). Tadpoles that found or accessed food faster after 24 hours of food deprivation were the largest (C). Differing symbols and trend lines are for replicate tanks.

Tadpoles that were initially of similar size and developmental stage when placed in groups stratified in body mass and Gosner stage in all replicate tanks by the end of the five-week experimental period. The mean mass gained by the smallest and largest tadpoles in each tank was 0.14±0.04 g (mean ± SEM) and 0.38±0.03 g, respectively. Mean Gosner stages for these same smallest and largest tadpoles were 35.3±0.7 and 37±0, respectively. Body mass at the end of the experiment was associated with growth rates (Figure 3A; Table 2; *F*_1,43.6_ = 93.9, *P*<0.0001). Faster growth rates were associated with greater mean foraging rates (Figure 3B; Table 2; *F*_1,42_ = 4.9, *P* = 0.03). Note that of the six replicate tanks four showed the expected positive correlations between foraging and growth (Pearson *r* mean ± SEM, 0.5±0.24) and two tanks had negative correlations (−0.39±0.27). Larger tadpoles also more quickly accessed food after 24 hrs of food deprivation in all replicate groups (Figure 3C; Table 2; *F*_1,14.8_ = 7.7, *P* = 0.01).

**Table 2:** F-statistics and P-values for the random-effects GLM of behaviors associated with the final body mass of tadpoles reared in groups.

	Final mass		
Source	DF	F Ratio	Prob > F
Forage mean	1, 39.2	0.4415	0.5103
Time to food	1, 39.3	4.1145	**0.0493**
Growth rate	1, 41.99	58.5926	**<0.0001**
Forage mean*Growth rate	1, 39.05	0.4247	0.5184

### Stress axis activity in experimental groups

**Figure 4:**
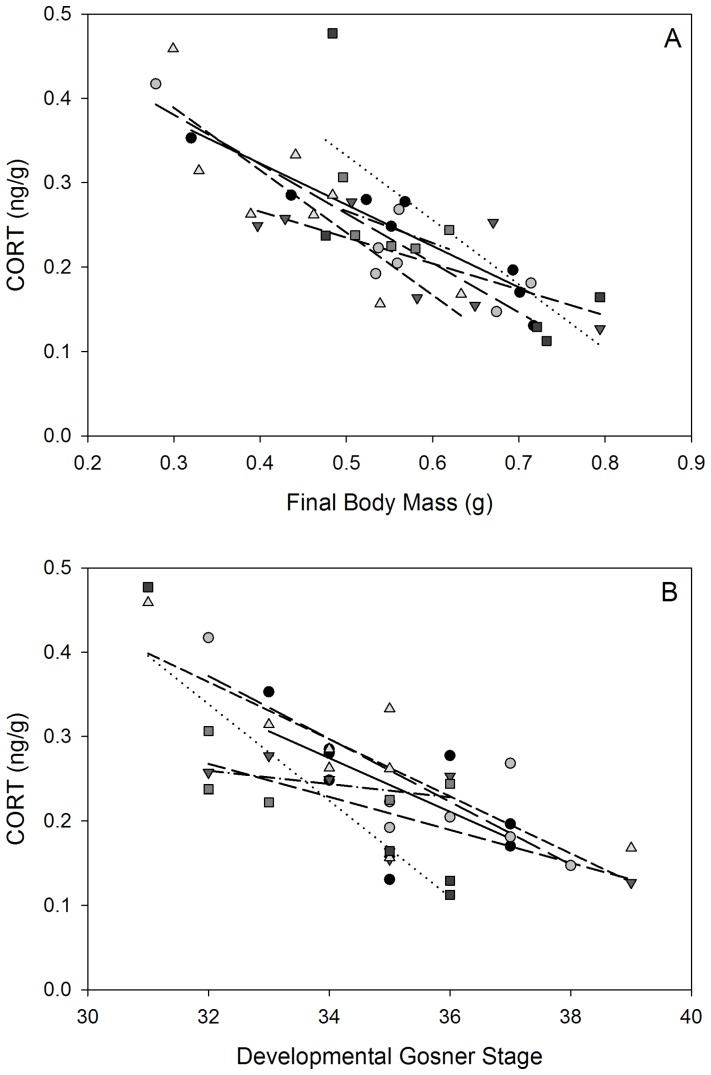
Patterns of corticosterone (CORT) concentration for tadpoles reared in replicate groups since hatching. CORT was associated with final body mass (A) and developmental Gosner stage (B) within replicate groups. Differing symbols and trend lines are for replicate tanks.

After experimental groups were established at hatching, tadpoles stratified by body size in which mean (± SEM) body mass at the time of collection for the smallest and largest tadpoles across replicate groups ranged from 0.38±0.04 g to 0.69±0.05 g and mean Gosner stages ranged from 32±0.4 to 37±0.8. CORT content was negatively associated with body mass (Figure 4A; Table 3; *F*_1,12.7_ = 26.2, *P*<0.001), with the smallest tadpoles having the highest CORT levels within each group. Based on the full GLM that included both body mass outweighed Gosner developmental stage, which did not have a significant association with CORT (Figure 4B; Table 3; *F*_1,21.4_ = 2.4, *P* = 0.14).

**Table 3:** F-statistics and P-values for the random-effects GLM of size and development associations with corticosterone (CORT) levels of tadpoles reared in groups.

	CORT		
Source	DF	F Ratio	Prob >F
Body mass	1, 12.7	26.2	**0.001**
Gosner stage	1, 21.4	2.34	0.138
Mass * Gosner Stage	1, 35.2	0.164	0.688

## Discussion

Our study demonstrated that size variation among similarly aged larval amphibians is generated through both physiological and behavioral differences between individuals. These individual traits were partly associated familial differences in growth rate, but more so by differences in their hatching date, which could result from variance in genetics, maternal effects, and environmental conditions experienced by eggs within a clutch. This is consistent with amphibian studies comparing populations which have shown that genetic variance to some extent underlies phenotypic variation in growth rates [11], [44]. However, within populations larval growth appears to be largely determined by environmental factors coupled with the influence of maternal effects [8], [11], [44], [45]. In amphibians, maternal effects can include egg size, clutch size and placement within a pond. Indeed, previous studies have highlighted the importance of egg size for determining hatchling size in amphibians [8], [18], [46], [47]. It is unclear what factors led to earlier/later hatching in our clutches but placement within the egg masses (interior vs. exterior) may have been important as eggs on the exterior appeared to have hatched the earliest (potentially due to greater dissolved gas exchange). In addition, environmental cues including hypoxia levels, pathogen exposure and predator cues have also been shown to be important factors contributing to plasticity in hatching patterns among amphibians [48]. However, plastic hatching responses to such cues have been linked to life history trade-offs between development, growth and survival across life stages [48]. Indeed, our finding that the earliest hatching tadpoles were initially the smallest but grew the most and developed the fastest also suggest that factors regulating these processes may impose life history trade-offs and influence the performance of individuals within varied environmental conditions.

Studies of birds have shown that hatching order can influence several phenotypic traits including body size and growth rates of hatchlings [49]–[51]. In addition, these studies have suggested that resource allocation to growth may trade-off with allocation to immune function [49] and influence long-term survival [50]. Given these processes, it is reasonable to expect that the early hatching and small-bodied tadpoles in our study that exhibited the fastest growth and development may have done so at the expense of other physiological functions like immunity. Indeed, larval catch-up growth can shape adult physiological function in amphibians and have lasting effects on stress responsiveness, disease susceptibility and survival [7], [52], [53]. Examining the effects of hatching order on such life history trade-offs will require future experimentation, but these results suggest that plasticity in hatching patterns may also shape size specific physiology and behavior throughout the life of individuals to influence population level interactions and processes.

Scaling these hatching and early life patterns to population level dynamics remains a challenge, but our behavioral assays of tagged tadpoles randomly mixed from the same egg clutches as above, suggest that foraging behaviors of larval amphibians influence their growth trajectories. In our study tadpoles that exhibited faster growth rates also exhibited tendencies to forage more [5] and to more readily access food; as shown by quicker accessing of food in large ranked tadpoles following a 24 hr food deprivation. The association between faster accessing of food in larger tadpoles may suggest that these tadpoles are better competitors for food than smaller tadpoles (Fig. 3D). Across many animal taxa and populations, size variance reflects the degree to which animals are competing for food, and in turn, body size often determines the outcome of competitive interactions [54], [55]. In anurans it has generally been difficult to demonstrate competitive interactions, but several studies have suggested that inter- and intraspecific exploitative competition is present in tadpoles under low resource or high density conditions [15]–[17], [24], [56]. Alternatively, variation in the performance of tadpoles in these behaviors may also reflect individual hunger levels (i.e., appetite), motivation to find food, or sensory ability to detect food. Regardless of the underlying factors driving the patterns in our study, two recent studies have also shown that behaviors in tadpoles are consistent and repeatable, even past metamorphosis [57], and can influence susceptibility to parasitic infections [58]. Taken together these findings suggest that while the importance of behavioral differences among tadpoles has generally been overlooked, such variance is likely fundamental to individual growth, development and performance [59]. The next step for future study requires linking individual behavioral differences to intrinsic factors like physiological stress responsiveness, genetics and maternal effects (e.g. hatching order/date); this could be feasible in tadpoles if they are individually marked shortly after hatching.

These differences in growth patterns are likely also influenced by responses to physiological stress imposed by density-dependent factors that could include competition and food limitation. Across animal taxa, physiological and behavioral responses to population dynamics are associated with consistent size-dependent differences in activity of the HPI axis [54]{Formatting Citation}{Formatting Citation}{Formatting Citation}. Consistent with studies in other vertebrates [21], [60] we found that smaller sized tadpoles in experimental groups had elevated baseline CORT profiles relative to larger and more developed tadpoles. Note that anuran tadpoles generally increase CORT levels as they near metamorphosis (Gosner stages 41–45 in wood frogs) [61], as CORT synergizes with thyroid hormone to drive morphogenic change at this time [33]. However, the tadpoles in our experiment were between Gosner stages 32–37, and thus we believe reflected density-dependent effects on HPI activity. Although we were not able to follow individuals in this experiment because they were too small to tag at hatching, the variance in body sizes after 8 weeks likely reflected density-dependent interactions on growth rates because these tadpoles were collected on a single day of hatching with very similar sizes and the same development stage. We do not know, however, if the smaller tadpoles with higher CORT initially had more active HPI axes from hatching (e.g. were genetically/ environmentally predisposed), or if group interactions induced chronically higher CORT levels in these tadpoles. Recent studies have shown, however, that visual cues of density are sufficient to elevate CORT levels and alter phenotypic traits and behavior in wood frog tadpoles [22], [23], [62]. Coupled with our behavioral experiment, these findings suggest that HPI axis activity at least contributes to the regulation of differential growth rates in tadpole populations. However, future experiments are needed to determine if smaller individuals are predisposed to exhibit elevated CORT levels and suppressed growth from hatching. Such dynamics are suggested by the “prior attributes” hypothesis which states that genetics, physiology and behavior inherent to individuals predispose them to specific responses to environmental conditions [63]. Alternatively, very small differences in traits among a hatching cohort (e.g. hatching time) may set the growth trajectory of individuals which is then further modulated by density dependent interactions.

In conclusion, our study provides insight into the physiological, behavioral and developmental factors that contribute to size variation among similarly aged larval amphibians. We found the timing of hatching to be a strong determinant of variance in growth rates, body size and metamorphic traits. So any factors that might regulate hatching time, such as maternal effects (e.g. egg size) could have long-term phenotypic effects on adult physiology and behavior. Indeed, in vertebrates including amphibians catch-up growth is associated with permanently altered HPI axis activity and may be related to differing coping styles to extrinsic stressors [60], [64]. Another intrinsic factor that may be related to these hatching time, development and growth patterns is sex [65]. Studies of birds have found hatching to differ among the sexes and to potentially be an adaptive maternal effect [49]–[51]. In tadpoles, we also recently found sex differences in development rates, growth rates and metamorphic traits [unpublished data]. While the findings in this current study shed light on factors that contribute to size variation in amphibians, these results also raise compelling questions regarding the extent to which maternal, genetic and other intrinsic factors shape larval and adult phenotypes.
